# Comparative Relatedness of *Clostridioides difficile* Strains Isolated from Humans and Companion Dogs in South Korea

**DOI:** 10.3390/antibiotics14121231

**Published:** 2025-12-06

**Authors:** Joo Yeol Kim, Su Min Kwak, Jae Hong Jeong, Jae Young Oh, Kwang-Won Seo, Dongheui An, Dokyun Kim, Seok Hoon Jeong, Chang-Ki Kim, Kwang Jun Lee, Jong-Chan Chae

**Affiliations:** 1 Division of Biotechnology, Jeonbuk National University, Iksan 54596, Republic of Korea; rlawnduf@jbnu.ac.kr (J.Y.K.); ksm1109@jbnu.ac.kr (S.M.K.); yjjj2002@jbnu.ac.kr (J.H.J.); ohjy1026@jbnu.ac.kr (J.Y.O.); 2 Advanced Institute of Environment and Bioscience, Jeonbuk National University, Iksan 54596, Republic of Korea; 3 College of Veterinary Medicine, Chungbuk National University, Cheongju 28644, Republic of Korea; vetskw16@chungbuk.ac.kr; 4 Seegene Medical Foundation, Seoul 04805, Republic of Korea; amorvie74@mf.seegene.com; 5 Department of Laboratory Medicine and Research Institute of Bacterial Resistance, Gangnam Severance Hospital, Yonsei University College of Medicine, Seoul 06273, Republic of Korea; kyunsky@yuhs.ac (D.K.); kscpjsh@yuhs.ac (S.H.J.); 6 Seoul Clinical Laboratories, Yongin 16954, Republic of Korea; changki_kim@scllab.co.kr; 7 Division of Zoonotic and Vector Borne Disease Research, National Institute of Health, Cheongju 28159, Republic of Korea

**Keywords:** *Clostridioides difficile*, antimicrobial resistance, toxin gene, whole-genome sequencing, companion dog

## Abstract

**Background/Objectives**: * Clostridioides difficile* is an anaerobic Gram-positive bacterium and a leading cause of healthcare-associated diarrhea. In this study, *C. difficile* strains isolated from human patients with diarrhea and companion dogs in South Korea were compared to reveal the potential transmission between different hosts. **Methods**: A total of 304 *C. difficile* strains were isolated, including 217 human isolates and 87 dog isolates. The strains were characterized for antimicrobial susceptibility and genotypic features, including antimicrobial resistant genes and toxin genes. In addition, comparative genomic analyses were performed to investigate their genetic relatedness. **Results**: Although antimicrobial susceptibility test revealed no significant difference in overall resistance, human isolates had higher resistance to moxifloxacin and cefotetan, while dog isolates showed slightly higher resistance to clindamycin and ampicillin. Resistance to vancomycin (3.7%), rifampin (8.3%), and chloramphenicol (0.9%) was observed only in human isolates. Toxin genes (*tcdA* and *tcdB*) were found in 57.1% of human isolates and 43.7% of dog isolates, while binary toxin genes (*cdtA* and *cdtB*) were detected only in isolates from humans. Multilocus sequence typing (MLST) analysis identified 34 sequence types (STs) in human isolates and 16 in dog isolates. Among them, 15 STs were detected in the isolates from both origins; notably, ST203 and ST42 were the predominant taxa that were equally derived from humans and dogs. Although *tcdA* and *tcdB* have not been previously reported in ST203, they were detected in 7 out of 34 ST203 isolates. The whole genomes of 36 representative isolates belonging to ST42 and ST203 were classified according to the STs of the source origin. **Conclusions**: These results indicate that similar *C. difficile* strain populations are present in both humans and companion dogs, which is compatible with interspecies dissemination or circulation of shared strain populations, and may also reflect host adaptation.

## 1. Introduction

*Clostridioides difficile* is a Gram-positive, spore-forming, and anaerobic bacterium commonly found in the intestines of humans, various animals, and the environment [1,2,3]. It is a well-known causative agent of antibiotic-associated diarrhea and pseudomembranous colitis in humans, particularly following the disruption of gut microbiota due to antibiotic treatment [2,3]. The clinical manifestations of *C. difficile* infection (CDI) range from mild diarrhea to severe colitis, toxic megacolon, and even fatal outcomes. While *C. difficile* has been extensively studied in humans, recent reports have demonstrated that it also plays a significant role in gastrointestinal diseases in animals, including companion animals such as dogs and cats [4,5]. Reports of CDI in pets raise concerns regarding zoonotic transmission and the potential for cross-species spread. The presence of identical or closely related *C. difficile* ribotypes in both humans and their pets further supports the hypothesis of interspecies transmission, which could have significant implications for public health [5].

Given the critical role of antibiotics in disrupting the gut microbiota and facilitating *C. difficile* colonization, antimicrobial resistance (AMR) has further complicated CDI management. Although metronidazole, vancomycin, and fidaxomicin remain the recommended therapeutic agents for CDI, the emergence of antibiotic-resistant *C. difficile* strains continues to pose significant clinical challenges [6,7]. In contrast, fluoroquinolones, clindamycin, and certain beta-lactams are not used to treat CDI but are well-recognized high-risk antibiotics that predispose individuals to infection by disrupting the normal gut microbiota. Their widespread use and increasing resistance trends underscore the importance of antimicrobial stewardship and preventive strategies rather than revisions of CDI treatment protocols [8,9]. Therefore, understanding the genetic and biochemical characteristics of antibiotic-resistant *C. difficile* is crucial for improving therapeutic approaches and enhancing infection control measures.

The pathogenicity of *C. difficile* is primarily mediated by two major exotoxins, toxin A (*tcdA*) and toxin B (*tcdB*), which disrupt the intestinal epithelial barrier, leading to colitis and diarrhea. The toxin genes (*tcdA* and *tcdB*) are found in the 19.6 kb pathogenicity locus (PaLoc), which also includes regulatory genes such as *tcdR*, *tcdC*, and *tcdE* responsible for the control of toxin expression and secretion [10,11]. Additionally, a binary toxin, *C. difficile* transferase (CDT) encoded by *cdtA* and *cdtB*, has been associated with strains classified as hypervirulent [10,12]. These hypervirulent strains often possess mutations in the *tcdC* gene, a negative regulator of toxin expression, leading to increased toxin production and heightened disease severity in both human and animal hosts [13]. Notably, hypervirulent strains such as *C. difficile* ribotype 027 [RT027, sequence type (ST) 1] and ribotype 078 (RT078, ST11) have been identified in both human and animal populations, supporting the hypothesis of interspecies transmission [14,15]. Understanding the molecular mechanisms associated with toxin expression and regulation is essential for designing effective therapeutic interventions and implementing improved infection control strategies.

The genetic background of *C. difficile* strains is one of the significant factors for investigating their epidemiology and virulence. Multilocus sequence typing (MLST) is a widely used molecular typing technique that provides high-resolution differentiation of bacterial isolates by analyzing sequence variations in seven conserved housekeeping genes (*adk*, *atpA*, *dxr*, *glyA*, *recA*, *sodA*, and *tpi*). This method has been applied to elucidate the evolutionary relationships, global distribution, and transmission pathways of *C. difficile* strains [16]. Moreover, MLST is used to elucidate the correlation of genetic variation with antimicrobial resistance patterns and pathogenic potential, making it a powerful tool for monitoring hypervirulent and drug-resistant strains in both human and animal reservoirs. The *C. difficile* strains belonging to ST1 and ST11 are recognized as hypervirulent, as they harbor mutations in a regulatory gene, *tcdC*, which are associated with increased toxin production [14,15,17]. These hypervirulent STs have been reported to be responsible for large-scale outbreaks in hospital settings and have been detected in both human and animal specimens, raising concerns regarding zoonotic transmission. Similarly, ST37 (ribotype 017, RT017) exhibits a unique toxin profile, *tcdA*-negative/*tcdB*-positive (*A*^−^*B*^+^), which may influence disease severity and therapeutic efficacy [18]. In addition to hypervirulent strains, ST2 (ribotype 014/020, RT014/020) is also commonly found in both hospital-acquired and community-associated *C. difficile* infections [14,19].

Despite increasing global evidence highlighting the zoonotic and One Health significance of *C. difficile*, a substantial knowledge gap persists in South Korea. Most Korean studies have focused exclusively on human clinical isolates, with limited data available on the colonization patterns, toxin gene profiles, antimicrobial resistance, and genomic diversity of *C. difficile* in companion animals. Consequently, the potential for interspecies transmission between humans and pets in Korea has not been systematically evaluated, despite international reports documenting shared ribotypes or sequence types between human and animal isolates [5,20,21,22,23].

In order to elucidate the potential of cross-species dissemination of *C. difficile* between humans and companion dogs, microbial relationship was assessed by comparing the phenotypic and genomic characteristics of *C. difficile* isolates from these two types of hosts.

## 2. Results

### 2.1. Antimicrobial Susceptibility of C. difficile Isolated from Humans and Dogs

A total of 304 *C. difficile* strains were isolated in South Korea. Among these, 217 strains were obtained from human patients with diarrhea, and 87 strains were isolated from companion dogs (Table S1). A minimum inhibitory concentration (MIC) assay for eleven antibiotics was conducted to evaluate the antibiotic resistance of the isolates. The resistance rates were observed as shown in Table 1, including imipenem (IMI) resistance in 34.1% of human isolates and 18.4% of dog isolates, moxifloxacin (MXF) resistance in 26.3% of human isolates and 5.7% of dog isolates, and cefotetan (TANS) resistance in 13.4% of human isolates and 3.4% of dog isolates. Higher resistance rates were observed in human isolates relative to dog isolates for imipenem (*p* = 0.008), moxifloxacin (*p* < 0.001), and cefotetan (*p* = 0.012). In contrast, resistance to clindamycin (CLI) was detected in 33.6% of human isolates and 35.6% of dog isolates, along with ampicillin (AMP) resistance in 16.1% of human isolates and 18.4% of dog isolates, tetracycline (TET) resistance in 6.5% of human isolates and 8.0% of dog isolates, and metronidazole (MRD) resistance in 6.0% of human isolates and 5.7% of dog isolates. Slightly higher resistance rates were observed in dog isolates for these antibiotics. Additionally, vancomycin (VAN, *p* = 0.11) and rifampin (RIF, *p* = 0.003) resistance were detected exclusively in 3.7% and 8.3% of human isolates, respectively, although vancomycin resistance was not statistically significant. In summary, while resistance patterns to most antibiotics were comparable between human and companion dog *C. difficile* isolates, resistance to imipenem, moxifloxacin, and cefotetan was significantly higher in human isolates.

### 2.2. Distribution of Toxin Genes

The distribution of toxin A (*tcdA*) and toxin B (*tcdB*), the major exotoxins responsible for colitis and diarrhea, as well as the binary toxin genes *cdtA* and *cdtB* in *C. difficile* isolates was investigated via PCR (Table 2). In the analysis of 217 *C. difficile* isolates from human patients, 57.1% (124 isolates) harbored both *tcdA* and *tcdB* genes, 24.4% (53 isolates) possessed only *tcdB*, and 1.4% (3 isolates) carried only *tcdA*. On the other hand, the combinations of *tcdB-cdtA-cdtB* and *tcdA-tcdB-cdtA-cdtB* were each found in 0.9% (two isolates), and *tcdA-cdtA-cdtB* was observed in 0.5% (one isolate) of the strains. While no toxin genes were detected in 49 isolates among 87 *C. difficile* isolates from companion dogs, 43.7% (38 isolates) possessed *tcdA-tcdB*. These results indicate that toxin-positive *C. difficile* strains were significantly more prevalent in human isolates than in dog isolates (85.3% vs. 43.7%, *p* < 0.001). Moreover, the binary toxin genes, *cdtA* and *cdtB*, were detected exclusively in human isolates, although at a very low frequency (5/217 vs. 0/87, *p* = 0.33) (Table 2 and Table S2). However, toxin-producing *C. difficile* strains were identified in isolates from both humans and dogs, indicating that toxigenic genotypes are not restricted to specific host species.

### 2.3. Analysis of Taxonomic Lineage

**Figure 1:**
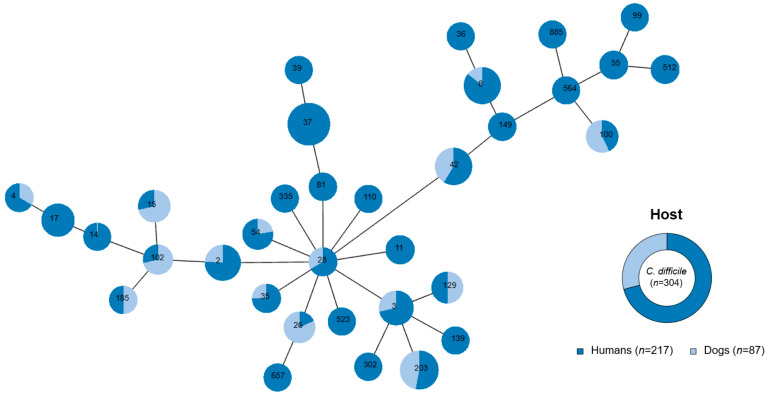
Minimum spanning tree (MST) of *C. difficile* strains from humans and dogs based on MLST. The MST illustrates the genetic relationships among 304 *C. difficile* isolates. Each node represents a distinct ST, with node size proportional to the frequency of each ST. The proportion of dark blue within each node indicates the number of isolates from humans, while the proportion of light blue represents the number of isolates from dogs.

In order to identify the taxonomic position of *C. difficile* isolates from humans and dogs, MLST was conducted with housekeeping genes (*adk*, *atpA*, *dxr*, *glyA*, *recA*, *sodA*, and *tpi*) (Figure 1 and Table S3). In human isolates, 34 different STs were identified, with ST37 being the most prevalent at 21.7% (47 isolates). On the other hand, 16 different STs were identified in dog isolates, and ST203 was the most dominant at 18.4% (16 isolates). While 19 STs were found exclusively in human isolates, 15 STs were identified in both human and dog isolates, including ST2, ST3, ST4, ST8, ST15, ST26, ST28, ST35, ST42, ST54, ST100, ST102, ST129, ST185, and ST203. These shared STs between humans and dogs indicate shared strain populations between the two hosts, possibly reflecting host-associated adaptation. Among them, the predominant shared ST was ST203, followed by ST42. To determine the taxonomic lineages among the *C. difficile* isolates based on the MLST results, a minimum spanning tree (MST) analysis was performed using PHYLOViZ (Figure 1). This analysis revealed a diverse ST network organized into five clonal complexes. Notably, ST28 was positioned at the center of the MST network, showing multiple connections with other STs. This result suggests its close genetic relationships with diverse lineages of *C. difficile*. Additionally, ST203, the most prevalent ST in dog isolates, was clustered with several STs shared between humans and dogs. This close genetic proximity suggests a recent divergence from a common ancestor or adaptation to similar environments. These findings indicate the substantial genetic diversity of *C. difficile* in humans and dogs, suggesting that certain STs are shared across host groups, potentially through common environmental exposure.

### 2.4. Genomic Relatedness of C. difficile Strains

**Figure 2:**
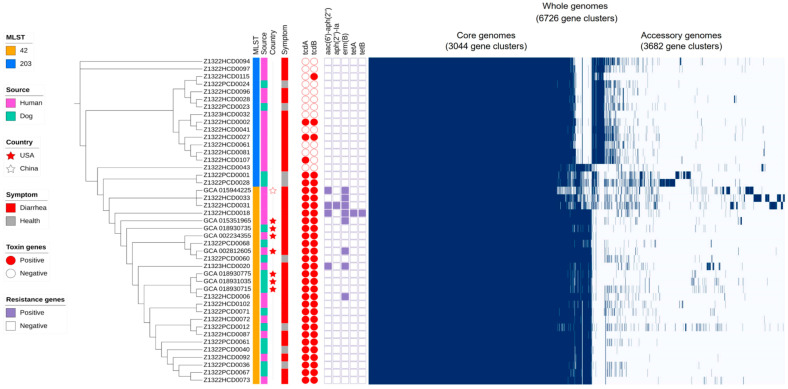
Genomic relatedness among 44 *C. difficile* strains from humans and dogs. Each row represents a single isolate, and the presence or absence of genes within the accessory genome is shown as blue lines. The cladogram on the left illustrates phylogenetic relationships among the isolates, with colored bars indicating sequence type (ST42: orange; ST203: blue), host origin (pink: human; teal: dog), and symptom status (red: diarrhea; gray: healthy). Panels indicate the presence (red) or absence (white) of toxin genes (*tcdA*, *tcdB*, *cdtA*, and *cdtB*) and the presence (purple) or absence (white) of antimicrobial resistance genes (*aac(6′)-aph(2″)*, *aph(2″)-Ia*, *erm(B)*, *tetA*, and *tetB*). Red and white stars denote the ST42 reference strains from the United States and China, respectively.

Among the genotypes of *C. difficile* commonly identified in both humans and dogs, we selected 36 isolates belonging to ST42 and ST203, the two most frequently shared lineages between the two hosts, for whole-genome sequencing. While ST2 and ST3 are globally recognized as representative toxigenic sequence types, ST42 is also a well-established toxigenic lineage frequently reported in both humans and animals. In this study, ST42 was detected more often in dogs and was included as a representative toxigenic sequence type. In contrast, ST203, which was also commonly detected in both hosts but had not previously been reported to carry toxin genes, was included to investigate its newly identified toxigenic characteristics. Therefore, comparative genomic analyses for ST42 and ST203 were subsequently conducted to explore potential epidemiological and phylogenetic associations between the human and dog isolates. The analyses included eight reference genomes of ST42 strains which have been reported and deposited in GenBank. The strains were isolated in the United States and China. However, no ST203 genome was available in public genome databases. The whole genomes of 44 *C. difficile* isolates from humans and dogs comprised a total of 6726 gene clusters, including 3044 core and 3682 accessory genes (Figure 2). The accessory genome showed a clear separation between ST42 and ST203, reflecting lineage-specific gene composition. Whole-genome comparison revealed two distinct genomic groups corresponding to sequence types ST42 and ST203, each including isolates from both hosts. Whereas ST203 comprised both *tcdA*/*tcdB*-positive and -negative strains, ST42 isolates including the reference strains consistently harbored both toxin genes (*tcdA* and *tcdB*). These toxin genes were also conserved among the reference ST42 strains despite minor variations in the accessory genome and antimicrobial resistance gene profiles. Although ST203 had not previously been reported to harbor toxin genes, our analysis identified strains carrying both *tcdA* and *tcdB* from humans and dogs, as well as strains carrying only *tcdA* or *tcdB* from humans (Figure 2 and Table S2). Antimicrobial resistance genes, including *aac(6′)-aph(2″)*, *aph(2″)-Ia*, *erm(B)*, *tetA*, and *tetB*, were mainly detected among ST42 isolates, while no *cdtA* or *cdtB* genes were found in any strain. Collectively, these findings suggest that *C. difficile* populations from humans and dogs are genetically distinct based on sequence type. Moreover, the detection of toxin genes in ST203 implies an expansion of toxigenic potential within this lineage, which had not previously been associated with pathogenicity.

### 2.5. Comparison of the tcdA and tcdB Regions

**Figure 3:**
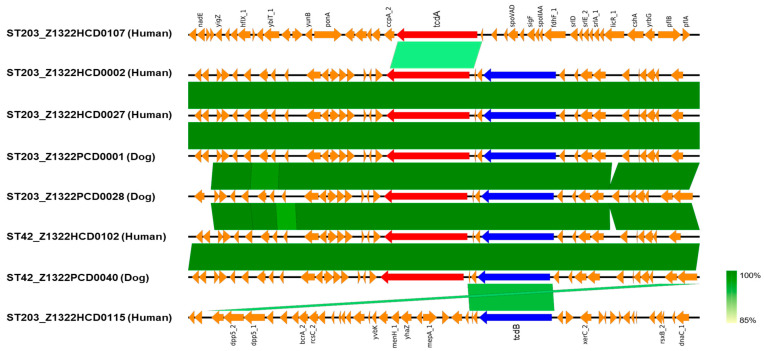
Comparative genomic alignment of the *tcdA* and *tcdB* regions in *C. difficile* ST42 and ST203 isolates. The alignment illustrates the structural organization and sequence similarity of the *tcdA* and *tcdB* regions between representative ST42 and ST203 genomes. Blue, red, and orange arrows correspond to *tcdA*, *tcdB*, and other coding sequences, respectively. Green shading represents nucleotide sequence identity (85–100%) between homologous regions, calculated using EasyFig (v.2.2.5).

Whole-genome analysis revealed that ST203 comprised both *tcdA*/*tcdB*-positive and -negative strains. To investigate genetic diversity, the *tcdA* and *tcdB* regions of representative ST42 and ST203 genomes were compared using EasyFig (Figure 3). The ST42 genome exhibited a typical toxin-producing configuration, with the *tcdA* and *tcdB* genes arranged consecutively within the PaLoc. In contrast, ST203 isolates displayed variable toxin-gene organizations: some carried both *tcdA* and *tcdB* in a structure nearly identical to that of ST42, whereas others possessed only a single toxin gene, suggesting structural variation within the corresponding locus. The high sequence similarity between ST42 and toxin-positive ST203 isolates indicates that the *tcdA* and *tcdB* regions in ST203 share an almost identical genetic structure with toxigenic lineages. In particular, ST42_Z1322HCD0102, which was isolated from humans, was found to be identical with ST203_Z1322HCD0002, ST203_Z1322HCD0027, and ST203_Z1322PCD0001 isolated from humans and dogs. These structural characteristics are consistent with the hypothesis that the PaLoc was acquired via horizontal gene transfer among ST203 isolates, although the direction of transfer cannot be determined from this study.

### 2.6. Genetic Relationships Among C. difficile Isolates Based on cgMLST Analysis

**Figure 4:**
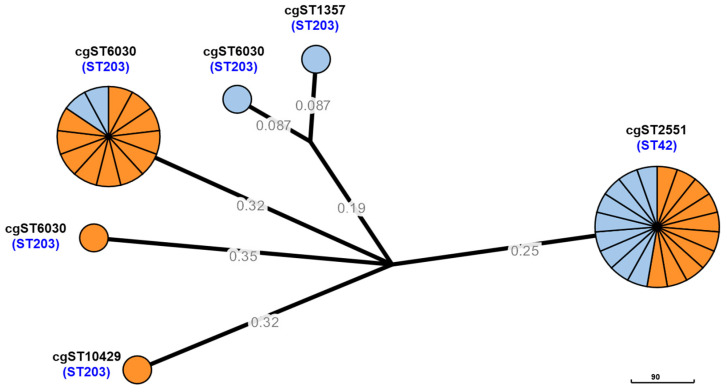
cgMLST analysis of 36 *C. difficile* strains from humans and dogs. Orange and sky blue represent *C. difficile* isolates from humans (*n* = 23) and dogs (*n* = 13), respectively. The scale bar indicates the branch length corresponding to sequence differences at 90 cgMLST loci.

To elucidate the phylogenetic relationships and genetic diversity of *C. difficile* isolates, a core genome multilocus sequence typing (cgMLST) analysis was conducted on 36 strains of ST42 and ST203 isolated from humans and dogs, as shown in Figure 4. The analysis revealed a variety of cgST types, with the major types being cgST2551 (ST42) and cgST6030 (ST203). Notably, cgST2551 (ST42) was identified in isolates from both humans and dogs, suggesting potential clonal expansion and host adaptation of *C. difficile* strains across these species. Additionally, the cgST6030 (ST203) clades also included isolates originating from both humans and dogs. These findings indicate the presence of genetically related shared strain populations between humans and dogs.

## 3. Discussion

*C. difficile* is increasingly recognized as a major pathogen in both humans and animals, causing a range of gastrointestinal illnesses from mild diarrhea to severe pseudomembranous colitis [20,24]. The increasing prevalence of *C. difficile* infections (CDIs) in healthcare environments, along with the growing number of community-acquired cases, has prompted the exploration of potential sources and transmission routes of this bacterium [21]. Recent studies have suggested that animals—particularly companion animals such as dogs—play a role in the dissemination of *C. difficile*, either as reservoirs or potential vectors for human infection [20,21]. In this study, we characterized *C. difficile* isolated from humans and dogs in South Korea and compared their genomes to explore potential interspecies dissemination. The antimicrobial resistance patterns of the *C. difficile* strains isolated from humans and dogs exhibited no significant difference, but human-derived isolates showed higher resistance to moxifloxacin (MXF, 26.3%, *p* < 0.001) and cefotetan (TANS, 13.4%, *p* = 0.012) compared with dog-derived isolates (MXF, 5.7%; TANS, 3.4%) (Table 1). These findings are consistent with previous reports indicating high resistance rates to fluoroquinolones and cephalosporins in clinical *C. difficile* isolates. The elevated resistance to moxifloxacin (MXF) and cefotetan (TANS) observed in this study supports the hypothesis that frequent antibiotic exposure in clinical settings acts as selective pressure, thereby facilitating the emergence and persistence of resistant strains [7,25]. Conversely, the slightly higher resistance rates observed in dog isolates for clindamycin (CLI) and ampicillin (AMP) suggest potential differences in antibiotic usage patterns in veterinary medicine [26,27]. Notably, vancomycin (VAN) and rifampin (RIF) resistance was exclusively identified in human isolates, suggesting that this resistance may be more closely associated with human clinical environments. As a result, the similarities in resistance patterns for most of the tested antibiotics may reflect shared strain populations or exposure to antibiotics belonging to the same classes. Nonetheless, variations in resistance to specific antibiotics likely reflect distinct selective pressures in human and veterinary environments. Antimicrobial resistance genes—*erm(B)* (clindamycin), *tetA*/*tetB* (tetracycline), and *aac(6′)-aph(2″)* (aminoglycosides)—were detected in ST42 isolates, as shown in Figure 2. The presence of these genes corresponded well with the phenotypic resistance profiles of the respective isolates. However, resistance to moxifloxacin and cefotetan, which was more common in human isolates, was not associated with identifiable resistance determinants, suggesting that chromosomal mutations or regulatory mechanisms may contribute to the phenotypes. Overall, the genomic AMR profiles demonstrated partial concordance with the observed MIC data, reflecting the involvement of both gene-mediated and non–gene-mediated resistance mechanisms.

Toxigenic *C. difficile* strains were more frequently detected in human isolates than in those from companion dogs, and the binary toxin genes (*cdtA* and *cdtB*) were exclusively identified in human-derived strains (Table 2). This observation is consistent with previous studies reporting a higher prevalence of toxigenic and binary toxin-positive *C. difficile* in humans compared with companion animals [5,17]. Such findings suggest that host-specific selective pressures, including clinical environments and antibiotic exposure, may contribute to the increased pathogenicity observed in human isolates [20,21,28]. In contrast, more than half of the companion dog isolates lacked toxin genes, which may indicate lower virulence or differences in colonization dynamics within dogs as a host. Although binary toxin genes were absent in companion dog isolates, over 40% of these strains were positive for both *tcdA* and *tcdB*. This suggests that companion dogs can serve as asymptomatic carriers of toxigenic strains commonly associated with human infection, raising concerns about sharing the same strain populations, especially in shared environments. The exclusive detection of *cdtA* and *cdtB* in human isolates further supports the idea that these virulence factors are more closely associated with clinical settings and human hosts [20,21,28]. Furthermore, previous studies have reported that genetically similar *C. difficile* ribotypes were isolated from humans and animals, including companion animals, and have raised the possibility of interspecies transmission [22,23]. These results underscore the need for a better understanding of the mechanisms underlying toxin gene acquisition and the epidemiological role of companion animals in the interspecies dissemination of toxigenic *C. difficile* strains.

In the MLST analysis, the identification of 34 STs in human isolates and 16 STs in dog isolates indicates the substantial genetic diversity of *C. difficile* (Figure 1 and Table S3). Among these, 15 STs were found to be shared between humans and dogs, with ST203 and ST42 as the isolates with relatively higher prevalence. This suggests the presence of shared strain populations, possibly arising from common environmental habitats or close contact, consistent with previous studies that have documented microbial genotype overlap between animal and human populations [22,23]. Additionally, MST analysis revealed that ST28 occupies a central position in the network, connecting multiple STs (Figure 1). This centrality suggests that ST28 may represent an ancestral or widely distributed lineage capable of adapting to diverse hosts. The presence of specific STs acting as pivotal nodes in phylogenetic networks indicates their evolutionary importance [29]. Until 2013, ST1—known to harbor toxin genes—was one of the predominant *C. difficile* types. However, between 2014 and 2020, new STs such as ST37, ST10, and ST203 emerged. Many of these STs were found to lack the *cdtA* gene, and ST203 is particularly known as a toxin-gene-deficient lineage, suggesting an evolutionary divergence from toxin-harboring lineages [17,28]. In this study, ST37 was the most abundant strain among human isolates, whereas ST203 was the most prevalent in dog isolates (Figure 1 and Table S3). In particular, several ST203 isolates from both humans and dogs harbored *tcdA*, *tcdB*, and *tcdA–tcdB* (Table S2). These results suggest the presence of shared strain populations in different hosts and adaptive variation among the same lineages. Therefore, the identification of ST203 in South Korea, which exhibits variation in toxin gene profiles, indicates the importance of continued surveillance.

After characterizing the antimicrobial resistance and toxin gene profiles of *C. difficile* isolates from humans and dogs, further genomic analyses were performed to investigate their phylogenetic relationships and genetic diversity. Based on the MLST and toxin gene profiles, ST42 and ST203 were selected as representative lineages for comparison of genomes (Figure 1 and Table S2). Among the genotypes identified as common in both humans and dogs, 36 isolates belonged to ST42 and ST203, which were the two most frequently shared lineages between the two hosts. Although ST2 and ST3 are globally recognized as representative toxigenic sequence types, ST42 is also a well-established toxigenic lineage frequently reported in both humans and animals. In this study, ST42 was detected more often in dogs and was therefore included as a representative toxigenic sequence type. In contrast, ST203—which was also commonly detected in both hosts but had not previously been reported to carry toxin genes—was included to investigate its newly identified toxigenic characteristics. The genome analysis of the 36 isolates belonging to ST42 and ST203 revealed distinct clusters in the pan-genome, core genome, and accessory genes (Figure 2). Specifically, the accessory genomes were more diverse and exhibited greater variation among isolates, being clearly classified according to sequence type. Whereas ST42 harbored the toxin genes *tcdA* and *tcdB*, ST203 displayed variability with respect to the presence of these toxin genes. Interestingly, although previous studies reported that ST203 lacks toxin genes [26], this study identified *tcdA*, *tcdB*, or *tcdA*-*tcdB* in isolates from both humans and dogs. This result indicates the genetic diversity of *C. difficile* and its capacity to acquire toxin genes, raising concerns about the emergence of novel pathogenic strains. Comparison of the *tcdA* and *tcdB* regions further revealed structural variation within the PaLoc among ST203 isolates (Figure 3). While ST42 exhibited the typical gene organization with *tcdA* and *tcdB* arranged consecutively, several ST203 isolates showed partial truncations or an absence of one of these genes. The high sequence similarity observed between toxin-positive ST203 and ST42 isolates is consistent with the hypothesis that ST203 may have acquired the PaLoc or its fragments via horizontal gene transfer. However, the directionality of this transfer cannot be determined solely from the genomic analysis. The transfer of the PaLoc in *C. difficile* was previously demonstrated by Hussain et al. [30] and supported by comparative genomic analyses, showing its mobility [31]. Previous reports have shown that PaLoc mobility can occur through homologous recombination at conserved flanking regions, integration of mobile genetic elements, or transposon-mediated exchange [30,31]. The partial *tcdA*/*tcdB* structures observed in ST203 imply such recombination-driven events. Toxin-gene acquisition and loss events may contribute to the ongoing evolution of *C. difficile* lineages, as evidenced by the emergence of novel toxigenic variants such as ST203. Additionally, cgMLST analysis provided further evidence of the relatedness of *C. difficile* populations from human and dog hosts (Figure 4), revealing shared genotypes (ST42 and ST203) that are present in both host species, thus supporting the potential for interspecies dissemination. As depicted in Figure 4, the phylogenetic clustering of isolates by sequence type indicated strong genetic relatedness within these lineages, regardless of the host origin. Overall, Overall, these results support the presence of shared strain populations between humans and dogs. Nevertheless, longitudinal or household-level epidemiological study would be required to elucidate the directionality of zoonosis between humans and dogs, together with the genomic insight obtained in this study.

## 4. Materials and Methods

### 4.1. Sample Collection and Isolation

Fecal samples were collected from 2083 human patients with diarrhea and 1405 pet dogs across eight regions of South Korea (Seoul, Gyeonggi, Incheon, Gangwon, Gyeongsang, Chungcheong, Jeolla, and Jeju) between 2022 and 2023 for the isolation of *C. difficile*. Each sample was suspended in 2 mL of buffered peptone water (Oxoid, Hampshire, UK), and 200 µL of the suspension was transferred to a cooked meat medium (Oxoid, UK). The samples were incubated under anaerobic conditions (10% CO_2_, 10% H_2_, 80% N_2_) at 37 °C for 48 h using an anaerobic jar and Anoxomat equipment (Advanced Instruments, Norwood, MA, USA). Following enrichment, colonies were sub-cultured for purification under anaerobic conditions at 37 °C for 48 h using ChromID *C. difficile* agar (CDIF) (BioMérieux, Marcy-l’Étoile, France) and blood agar (Synergy Innovation, Seongnam, Republic of Korea). The isolates were preserved in 10% Difco™ Skim Milk (BD, Franklin Lakes, NJ, USA) and stored at −80 °C.

### 4.2. DNA Extraction and Multiplex PCR for Toxin Genes

Genomic DNA was extracted using the LaboPass™ Bacteria Mini DNA purification kit (Cosmo Genetech, Seoul, Republic of Korea). The identification of *C. difficile* and the detection of its toxin genes were confirmed by matrix-assisted laser desorption/ionization time-of-flight (MALDI-TOF) mass spectrometry (Bruker, Bremen, Germany) and PCR, as previously described [32]. PCR reactions were performed using EzPCR™ HS 5× Premix (Elpis Biotech, Daejeon, Republic of Korea), with a total volume of 25 µL, consisting of 1 µL of extracted DNA and 10 pM of forward and reverse primers each (Table S4). The PCR conditions were as follows: initial denaturation at 93 °C for 2 min, followed by 30 cycles of 93 °C for 30 s, 60 °C for 1 min, and 68 °C for 1 min, with a final extension at 68 °C for 10 min. PCR amplicons were visualized via electrophoresis on a 1.5% agarose gel containing GreenLight™ Safe Gel Stain (BioAssay Co., Daejeon, Republic of Korea).

### 4.3. Multilocus Sequence Typing

MLST analysis was performed for all *C. difficile* strains using the PubMLST web portal (https://pubmlst.org/organisms/clostridioides-difficile, accessed on 20 May 2025). Seven housekeeping genes (*adk*, *atpA*, *dxr*, *glyA*, *recA*, *sodA*, and *tpi*) were amplified by PCR (Table S4), and the products were sequenced by a commercial provider (BioFact, Daejeon, Republic of Korea). The STs were assigned based on the PubMLST *C. difficile* database, and the taxonomic relationships among isolates were visualized by constructing an MST using PHYLOViZ version 2.0 (https://online.phyloviz.net/, accessed on 30 May 2025).

### 4.4. Antimicrobial Susceptibility Testing

Antimicrobial susceptibility was accessed using the broth microdilution method for a total of 11 antibiotics (ampicillin, cefotetan, clindamycin, imipenem, chloramphenicol, tetracycline, moxifloxacin, piperacillin/tazobactam, metronidazole, vancomycin, and rifampin). The turbidity of the bacterial suspension was adjusted to a 0.5 McFarland standard using a densitometer (BioMérieux, France) in 2 mL of Mueller–Hinton broth supplemented with TES buffer (Thermo Fisher Scientific, Waltham, MA, USA). Then, 100 µL of the adjusted suspension was inoculated into 11 mL of Sensititre Supplemented Brucella Broth (Oxoid, UK) containing 5% laked horse blood (Oxoid, UK). Subsequently, 100 µL of the prepared inoculum was dispensed into a customized Sensititre plate (KRCPECDI) (Thermo Fisher Scientific, USA). The plate was incubated under anaerobic conditions (10% CO_2_, 10% H_2_, 80% N_2_) at 37 °C for 48 h. The susceptibility results were interpreted according to the Clinical and Laboratory Standards Institute (CLSI M100) and European Committee on Antimicrobial Susceptibility Testing (EUCAST v.13.0) guidelines [33,34].

### 4.5. Whole-Genome Sequencing and Analysis

Among the total 63 isolates belonging to ST42 (*n* = 29; 17 human and 12 dog isolates) and ST203 (*n* = 34; 18 human and 16 dog isolates), 36 representative strains were selected for whole-genome sequencing. Selection was based on host origin, geographic region, sampling year, toxin gene profiles (*tcdA* and *tcdB*), antimicrobial resistance phenotypes, and the presence of AMR genes, ensuring maximal genomic, phenotypic, and epidemiological diversity. Whole-genome sequencing was performed using two sequencing platforms: the iSeq 100 (Illumina, San Diego, CA, USA) and the MinION (Oxford Nanopore Technologies, Oxford, UK). For the iSeq platform, libraries were prepared using the Illumina DNA Prep kit and Nextera DNA CD Indexes (Illumina, USA). For the MinION platform, libraries were generated using the Ligation Sequencing kit (SQK-LSK-109), Native Barcoding Expansion (EXP-NBD-104) (Oxford Nanopore Technologies, UK), and the NEBNext Companion Module (New England Biolabs, Ipswich, MA, USA). Sequencing on the MinION was conducted using an R9.4.1 flow cell (Oxford Nanopore Technologies, UK). Raw short-read sequences from the iSeq platform were processed using Trimmomatic v0.39 [35] to remove adapter sequences, primer contaminants, and low-quality reads. For raw long-read sequences generated by MinION, Filtlong v0.2.0 was used to filter low-quality reads, and Porechop v0.2.4 was employed for adapter trimming. De novo hybrid genome assembly with short- and long-read data was performed using Unicycler v0.4.9b [36]. Pan-genome analysis was conducted using Roary v3.12.0 [37], followed by genome annotation using Prokka v1.14.6 [38], which generated GFF3 files for downstream analysis. Comparative genomic analysis of the toxin gene-carrying strains (*tcdA* and *tcdB*) was performed using EasyFig (v2.2.5, Windows version) [39]. GenBank (.gbk) files generated by Prokka were used as input files, and the contig lengths containing the toxin loci were manually adjusted for each genome to visualize gene structures. Core genome phylogenetic relationships were inferred using IQ-TREE v2.0.3 [40] based on concatenated core gene alignments. The resulting phylogenetic tree was visualized and annotated with metadata, including MLST profiles and the presence/absence of toxin genes, using the Interactive Tree of Life (iTOL) [41]. For cgMLST, protein sequences were extracted using Prodigal v2.6.3 [42] and processed with chewBBACA v3.3.1 [43] to define cgMLST loci. Genetic relationships among isolates were further examined using a minimum spanning tree approach, visualized with GrapeTree [44], to assess genomic clustering and potential epidemiological links.

### 4.6. Statistical Analysis

Statistical analyses were performed using GraphPad Prism version 9.0 (GraphPad Software, San Diego, CA, USA). Categorical variables, including antibiotic resistance rates, toxin gene frequencies, and sequence type distributions between human- and dog-derived isolates, were compared using the chi-square test or Fisher’s exact test when appropriate. A *p*-value of <0.05 was considered statistically significant. 

## Figures and Tables

**Table 1:** Antimicrobial resistance of *C. difficile* isolates.

Antibiotics ^a^	Resistance Breakpoints Followed by CLSI/EUCAST	No. (%) of Resistance Strains	
		Humans (*n* = 217)	Dogs (*n* = 87)
AMP	≥2	35 (16.1)	16 (18.4)
MXF	≥8	57 (26.3)	5 (5.7)
VAN	≥2	8 (3.7)	0 (0.0)
P/T4	≥64/4	0 (0.0)	1(1.1)
CLI	≥8	73 (33.6)	31 (35.6)
RIF	≥4	18 (8.3)	0 (0.0)
MRD	≥2	13 (6.0)	5 (5.7)
TET	≥16	14 (6.5)	7 (8.0)
TANS	≥64	29 (13.4)	3 (3.4)
IMI	≥16	74 (34.1)	16 (18.4)
CHL	≥32	2 (0.9)	0 (0.0)

^a^ Antibiotics: AMP, ampicillin; MXF, moxifloxacin; VAN, vancomycin; P/T4, piperacillin/tazobactam constant 4; CLI, clindamycin; RIF, rifampin; MRD, metronidazole; TET, tetracycline; TANS, cefotetan; IMI, imipenem; CHL, chloramphenicol. *p*-values (human vs. dog, Fisher’s exact test): MXF (*p* < 0.001), TANS (*p* = 0.012), IMI (*p* = 0.008), RIF (*p* = 0.003), and others (*p* > 0.05).

**Table 2:** Distribution of *C. difficile* strains with toxin genes.

Subject	No. (%) of *C. difficile* with Toxin Genes					
	*tcdA*	*tcdB*	*tcdA-tcdB*	*tcdA-*	*tcdB-*	*tcdA-tcdB-*
				*cdtA-cdtB*	*cdtA-cdtB*	*cdtA-cdtB*
Humans (*n* = 217)	3 (1.4)	53 (24.4)	124 (57.1)	1 (0.5)	2 (0.9)	2 (0.9)
Dogs (*n* = 87)	-	-	38 (43.7)	-	-	-
Total (*n* = 304)	3 (1.0)	53 (17.4)	162 (53.3)	1 (0.3)	2 (0.7)	2 (0.7)

All *C. difficile* isolates were obtained from human patients with diarrhea, while 34.5% (30/87) of the isolates from companion dogs originated from animals exhibiting diarrheal symptoms. “-” indicates no isolates detected with the corresponding toxin gene combination. *p*-values (human vs. dog): *tcdA*-*tcdB* (*p* = 0.042), *tcdB* (*p* < 0.0001), and others *p* > 0.05.

## Data Availability

The genome sequences obtained in this study have been deposited in GenBank database under accession numbers CP149689-CP149766. Individual accession numbers for all 36 *C. difficile* isolates are provided in Supplementary Table S5.

## Supplementary Materials

The following supporting information can be downloaded at: https://www.mdpi.com/article/10.3390/antibiotics14121231/s1, Table S1: Regional distribution of *C. difficile* isolated from humans and dogs; Table S2: Distribution of *C. difficile* strains with toxin genes based on sequence type (ST); Table S3: Frequency of sequence types (STs) of *C. difficile* isolates from humans and dogs; Table S4: List of primers used in this study; Table S5. Strain information and GenBank accession numbers of 36 whole-genome sequences for *C. difficile* ST42 and ST203 isolates.

## Abbreviations

The following abbreviations are used in this manuscript:

AMR	Antimicrobial resistance
CDIs	*Clostridioides difficile* infections
MLST	Multilocus sequence typing
cgMLST	Core genome multilocus sequence typing
ST	Sequence type
WGS	Whole-genome sequencing
PaLoc	Pathogenicity locus

## References

[1] Wang X., Wang W.-Y., Yu X.-L., Chen J.-W., Yang J.-S., Wang M.-K. (2025). Comprehensive review of *Clostridium difficile* infection: Epidemiology, diagnosis, prevention, and treatment. World J. Gastrointest. Pharmacol. Ther..

[2] Darkow A., Boreyko J., Patel M. (2024). *Clostridioides difficile* Infection: A Review of Emerging Practices for Infection Treatment and Prevention of Recurrence. J. Transl. Gastroenterol..

[3] Clarke L.M., Allegretti J.R. (2024). Review article: The epidemiology and management of *Clostridioides difficile* infection—A clinical update. Aliment. Pharmacol. Ther..

[4] Salvarani F.M., Oliveira H.G.d.S., Uzal F.A. (2025). *Clostridioides difficile* in Animal Inflammatory Bowel Disease: A One Health Perspective on Emerging Zoonotic Threats. Microorganisms.

[5] Redding L.E., Habing G.G., Tu V., Bittinger K.L., O’Day J., Pancholi P., Wang S.-H., Alexander A., Kelly B.J., Weese J.S. (2023). Infrequent intrahousehold transmission of *Clostridioides difficile* between pet owners and their pets. Zoonoses Public Health.

[6] Wickramage I., Spigaglia P., Sun X. (2021). Mechanisms of antibiotic resistance of *Clostridioides difficile*. J. Antimicrob. Chemother..

[7] Dang Z., Yang B., Xia P., Huang J., Liao J., Li Y., Tang S., Han Q., Luo S., Xia Y. (2024). Antimicrobial susceptibilities, resistance mechanisms and molecular characteristics of toxigenic *Clostridioides difficile* isolates in a large teaching hospital in Chongqing, China. J. Glob. Antimicrob. Resist..

[8] Gilboa M., Regev-Yochay G., Meltzer E., Cohen I., Peretz Y., Zilberman-Daniels T., Segev A., Amit S., Yahav D., Barda N. (2025). Antibiotic use and the risk of hospital-onset *Clostridioides difficile* infection. JAMA Netw. Open.

[9] Salvati F., Catania F., Murri R., Fantoni M., Torti C. (2024). *Clostridioides difficile* infection: An update. Infez. Med..

[10] Alam M.Z., Madan R. (2024). *Clostridioides difficile* Toxins: Host Cell Interactions and Their Role in Disease Pathogenesis. Toxins.

[11] Majumdar A., Pillai S.K. (2022). Regulation of *Clostridioides difficile* toxin production. Curr. Opin. Microbiol..

[12] Simpson M., Bilverstone T., Leslie J., Donlan A., Uddin M.J., Petri W.A., Marin N., Kuehne S., Minton N.P., Petri W.A. (2023). *Clostridioides difficile* binary toxin binding component increases virulence in a hamster model. Open Forum Infect. Dis..

[13] Markovska R., Dimitrov G., Gergova R., Boyanova L. (2023). *Clostridioides difficile*, a New “Superbug”. Microorganisms.

[14] Weese J.S. (2020). *Clostridium* (*Clostridioides*) *difficile* in animals. J. Vet. Diagn. Investig..

[15] Alexiou S., Diakou A., Kachrimanidou M. (2025). The Role of *Clostridioides difficile* Within the One Health Framework: A Review. Microorganisms.

[16] Abad-Fau A., Sevilla E., Martín-Burriel I., Moreno B., Bolea R. (2023). Update on commonly used molecular typing methods for *Clostridioides difficile*. Microorganisms.

[17] Schüler M.A., Riedel T., Overmann J., Daniel R., Poehlein A. (2024). Comparative genome analyses of clinical and non-clinical *Clostridioides difficile* strains. Front. Microbiol..

[18] Li C., Heuler J., Zhu D., Meng X., Chakraborty S., Harmanus C., Wang S., Peng Z., Smits W.K., Wu A. (2024). Genomic and phenotypic characterization of a *Clostridioides difficile* strain of the epidemic ST37 type from China. Front. Cell. Infect. Microbiol..

[19] Miles-Jay A., Young V.B., Pamer E.G., Savidge T.C., Kamboj M., Garey K.W., Snitkin E.S. (2021). A multisite genomic epidemiology study of *Clostridioides difficile* infections in the USA supports differential roles of healthcare versus community spread for two common strains. Microb. Genom..

[20] Bjöersdorff O.G., Lindberg S., Kiil K., Persson S., Guardabassi L., Damborg P. (2021). Dogs are carriers of *Clostridioides difficile* lineages associated with human community-acquired infections. Anaerobe.

[21] Hernandez B.G., Vinithakumari A.A., Sponseller B., Tangudu C., Mooyottu S. (2020). Prevalence, colonization, epidemiology, and public health significance of *Clostridioides difficile* in companion animals. Front. Vet. Sci..

[22] Redding L.E., Tu V., Abbas A., Alvarez M., Zackular J.P., Gu C., Bushman F.D., Kelly D.J., Barnhart D., Lee J.J. (2022). Genetic and phenotypic characteristics of *Clostridium* (*Clostridioides*) *difficile* from canine, bovine, and pediatric populations. Anaerobe.

[23] Alves F., Castro R., Pinto M., Nunes A., Pomba C., Oliveira M., Silveira L., Gomes J.P., Oleastro M. (2023). Molecular epidemiology of *Clostridioides difficile* in companion animals: Genetic overlap with human strains and public health concerns. Front. Public Health.

[24] Sholeh M., Kouhsari E., Talebi M., Hallajzadeh M., Godarzi F., Amirmozafari N. (2021). Toxin gene profiles and antimicrobial resistance of *Clostridioides difficile* infection: A single tertiary care center study in Iran. Iran. J. Microbiol..

[25] Silva S.Y., Wilson B.M., Redmond S.N., Donskey C.J. (2021). Inpatient fluoroquinolone use in Veterans’ Affairs hospitals is a predictor of *Clostridioides difficile* infection due to fluoroquinolone-resistant ribotype 027 strains. Infect. Control Hosp. Epidemiol..

[26] Hur B.A., Hardefeldt L.Y., Verspoor K.M., Baldwin T., Gilkerson J.R. (2020). Describing the antimicrobial usage patterns of companion animal veterinary practices; free text analysis of more than 4.4 million consultation records. PLoS ONE.

[27] Wen G.-L., Li S.-H., Qin Z., Yang Y.-J., Bai L.-X., Ge W.-B., Liu X.-W., Li J.-Y. (2022). Isolation, molecular typing, and antimicrobial resistance of *Clostridium difficile* in dogs and cats in Lanzhou City of Northwest China. Front. Vet. Sci..

[28] Sholeh M., Beig M., Kouhsari E., Rohani M., Katouli M., Badmasti F. (2025). Global insights into the genome dynamics of *Clostridioides difficile* associated with antimicrobial resistance, virulence, and genomic adaptations among clonal lineages. Front. Cell. Infect. Microbiol..

[29] Pecora N., Holzbauer S., Wang X., Gu Y., Taffner S., Hatwar T., Hardy D., Dziejman M., D’Heilly P., Pung K. (2022). Genomic analysis of *Clostridioides difficile* in two regions of the United States reveals a diversity of strains and limited transmission. J. Infect. Dis..

[30] Hussain H., Nubgan A., Rodríguez C., Imwattana K., Knight D.R., Parthala V., Mullany P., Goh S. (2024). Removal of mobile genetic elements from the genome of *Clostridioides difficile* and the implications for the organism’s biology. Front. Microbiol..

[31] Tickler I.A., Goering R.V., Tenover F.C. (2025). History and evolution of the hypervirulent *Clostridioides difficile* ribotype 027 lineage. Microorganisms.

[32] Chankhamhaengdecha S., Hadpanus P., Aroonnual A., Ngamwongsatit P., Chotiprasitsakul D., Chongtrakool P., Janvilisri T. (2013). Evaluation of multiplex PCR with enhanced spore germination for detection of *Clostridium difficile* from stool samples of hospitalized patients. BioMed Res. Int..

[33] Clinical and Laboratory Standards Institute (CLSI) (2024). Performance Standards for Antimicrobial Susceptibility Testing.

[34] The European Committee on Antimicrobial Susceptibility Testing (EUCAST) (2023). Breakpoint Tables for Interpretation of MICs and Zone Diameters, Version 13.0.

[35] Bolger A.M., Lohse M., Usadel B. (2014). Trimmomatic: A flexible trimmer for Illumina sequence data. Bioinformatics.

[36] Wick R.R., Judd L.M., Gorrie C.L., Holt K.E. (2017). Unicycler: Resolving bacterial genome assemblies from short and long sequencing reads. PLoS Comput. Biol..

[37] Page A.J., Cummins C.A., Hunt M., Wong V.K., Reuter S., Holden M.T.G., Fookes M., Falush D., Keane J.A., Parkhill J. (2015). Roary: Rapid large-scale prokaryote pan-genome analysis. Bioinformatics.

[38] Seemann T. (2014). Prokka: Rapid prokaryotic genome annotation. Bioinformatics.

[39] Sullivan M.J., Petty N.K., Beatson S.A. (2011). Easyfig: A genome comparison visualizer. Bioinformatics.

[40] Nguyen L.T., Schmidt H.A., von Haeseler A., Minh B.Q. (2015). IQ-TREE: A fast and effective stochastic algorithm for estimating maximum-likelihood phylogenies. Mol. Biol. Evol..

[41] Letunic I., Bork P. (2007). Interactive Tree of Life (iTOL): An online tool for phylogenetic tree display and annotation. Bioinformatics.

[42] Hyatt D., Chen G.L., LoCascio P.F., Land M.L., Larimer F.W., Hauser L.J. (2010). Prodigal: Prokaryotic gene recognition and translation initiation site identification. BMC Bioinform..

[43] Silva M., Machado M.P., Silva D.N., Rossi M., Moran-Gilad J., Ramirez M. (2018). chewBBACA: A complete suite for gene-by-gene schema creation and strain identification. Microb. Genom..

[44] Zhou Z., Alikhan N.-F., Sergeant M.J., Luhmann N., Vaz C., Francisco A.P., Carriço J.A., Achtman M. (2018). GrapeTree: Visualization of core genomic relationships among 100,000 bacterial pathogens. Genome Res..

