# Supplementary material for: Comparative Relatedness of Clostridioides difficile Strains Isolated from Humans and Companion Dogs in South Korea
Source: Antibiotics (Basel). 2025 Dec 6;14(12):1231. doi: 10.3390/antibiotics14121231 (PMC12729887; doi:10.3390/antibiotics14121231)
Supplement: Supplementary file 1 [file antibiotics-14-01231-s001.zip › antibiotics-3989451-supplementary.pdf]

Table S1. Regional distribution of *Clostridioides difficile* isolated from humans and dogs.

Origin			No. (%) of isolates of *C. difficile* by region								Total
			SE[a]	GG	IC	GW	GS	CC	JE	JJ	
Humans	Gender	Female	10 (8.8)	36 (31.6)	18 (15.8)	1 (0.9)	27 (23.7)	1 (0.9)	20 (17.5)	1 (0.9)	114 (52.5)
		Male	6 (5.8)	39 (37.9)	15 (14.6)	2 (1.9)	17 (16.5)	3 (2.9)	21 (20.4)	-	103 (47.5)
	Age	0-10	-	17 (17.3)	22 (22.4)	-	25 (25.5)	1 (1.0)	33 (33.7)	-	98 (45.2)
		11-20	-	2 (33.3)	1 (16.7)	1 (16.7)	2 (33.3)	-	-	-	6 (2.8)
		21-30	-	-	2 (66.7)	-	1 (33.3)	-	-	-	3 (1.4)
		31-40	-	3 (75.0)	-	-	1 (25.0)	-	-	-	4 (1.8)
		41-50	-	2 (100)	-	-	-	-	-	-	2 (0.9)
		51-60	3 (23.1)	6 (46.2)	1 (7.7)	-	-	1 (7.7)	2 (15.4)	-	13 (6.0)
		61-70	1 (6.7)	5 (33.3)	3 (20.0)	-	4 (26.7)	-	2 (13.3)	-	15 (6.9)
		71-80	7 (23.3)	15 (50.0)	2 (6.7)	1 (3.3)	2 (6.7)	-	3 (10.)	-	30 (13.8)
		81-90	5 (12.8)	20 (51.3)	2 (5.1)	1 (2.6)	7 (17.9)	2 (5.1)	1 (2.6)	1 (2.6)	39 (18.0)
		91-100	-	5 (71.4)	-	-	2 (28.6)	-	-	-	7 (3.2)
	Subtotal		16 (7.4)	75 (34.6)	33 (15.2)	3 (1.4)	44 (20.3)	4 (1.8)	41 (18.9)	1 (0.5)	217
Dogs			67 (77.0)	8 (9.2)	NP[b]	NP	NP	12 (13.8)	NP	NP	87

[a] SE, Seoul; GG, Gyeonggi; IC, Incheon; GW, Gangwon; GS, Gyeongsang; CC, Chungcheong; JE, Jeolla; JJ, Jeju, [b] Not performed. "–" indicates no isolates detected in the corresponding region or category

Table S2. Distribution of *Clostridioides difficile* strains with toxin genes based on sequence type (ST).

MLST[a]	No. of *C. difficile* with toxin genes					
	tcdA		*tcdB*		*tcdA-tcdB*	
	Humans	Dogs	Humans	Dogs	Humans	Dogs
ST2 (n=25)	-	-	-	-	19	6
ST3 (n=21)	-	-	-	-	11	-
ST4 (n=3)	-	-	-	-	2	1
ST8 (n=27)	-	-	3	-	21	3
ST14 (n=1)	-	-	-	-	1	-
ST17 (n=16)	-	-	-	-	16	-
ST28 (n=3)	1	-	-	-	-	-
ST35 (n=4)	-	-	-	-	3	1
ST36 (n=1)	-	-	-	-	1	-
ST37 (n=47)	-	-	45	-	2	-
ST42 (n=29)	-	-	-	-	17	12
ST54 (n=9)	-	-	-	-	7	2
ST55 (n=3)	-	-	-	-	3	-
ST81 (n=2)	-	-	2	-	-	-
ST99 (n=1)	-	-	-	-	1	-
ST100 (n=14)	-	-	2	-	-	-
ST102 (n=7)	-	-	-	-	2	4
ST110 (n=1)	-	-	-	-	1	-
ST129 (n=12)	-	-	-	-	6	6
ST139 (n=1)	-	-	-	-	1	-
ST149 (n=2)	-	-	-	-	2	-
ST185 (n=2)	-	-	-	-	1	1
ST203 (n=34)	2	-	1	-	2	2
ST302 (n=1)	-	-	-	-	1	-
ST512 (n=3)	-	-	-	-	3	-
ST567 (n=1)	-	-	-	-	1	-

[a] STs carrying toxin genes in human and/or dog isolates; non-toxigenic STs are not shown.

″–″ indicates no isolates detected for the corresponding toxin gene.

Rare combinations with *cdtA–cdtB* (n=5) were detected only in human isolates (one ST885 strain with *tcdA–cdtA–cdtB* and two ST11 strains with either *tcdB–cdtA–cdtB* or *tcdA–tcdB–cdtA–cdtB*).

Table S3. Frequency of sequence types (STs) of *C. difficile* isolates from humans and dogs.

Sequence type (ST)	No. (%) of *C. difficile*	
	Humans (n=217)	Dogs (n=87)
2	19 (8.8)	6 (6.9)
3	15 (6.9)	6 (6.9)
4	2 (0.9)	1 (1.1)
8	24 (11.1)	3 (3.4)
11	4 (1.8)	-
14	1 (0.5)	-
15	4 (1.8)	10 (11.5)
17	16 (7.4)	-
26	2 (0.9)	8 (9.2)
28	2 (0.9)	1 (1.1)
35	3 (1.4)	1 (1.1)
36	1 (0.5)	-
37	47 (21.7)	-
39	1 (0.5)	-
42	17 (7.8)	12 (13.8)
54	7 (3.2)	2 (2.3)
55	3 (1.4)	-
81	2 (0.9)	-
99	1 (0.5)	-
100	6 (2.8)	8 (9.2)
102	2 (0.9)	5 (5.7)
110	1 (0.5)	-
129	6 (2.8)	6 (6.9)
139	1 (0.5)	-
149	2 (0.9)	-
185	1 (0.5)	1 (1.1)
203	18 (8.3)	16 (18.4)
239	-	1 (1.1)
302	1 (0.5)	-
335	1 (0.5)	-
512	3 (1.4)	-
523	1 (0.5)	-
564	1 (0.5)	-
657	1 (0.5)	-
885	1 (0.5)	-

"–" indicates no isolates detected.

Table S4. List of primers used in this study.

Target gene	Primer sequence (5'-3')	Product size (bp)
16S rRNA	F- GCCTAACACATGCAAGTCGA	800
	R- TACCAGGGTATCTAATCC	
tcdA	F- GTATGGATAGGTGGAGAAGTCAGTG	632
	R- CGGTCTAGTCCAATAGAGCTAGGTC	
tcdB	F- GAAGATTTAGGAAATGAAGAAGGTGA	441
	R- AACCACTATATTCAACTGCTTGTCC	
cdtA	F- ATGCACAAGACTTACAAAGCTATAGTG	260
	R- CGAGAATTTGCTTCTATTTGATAATC	
cdtB	F- ATTGGCAATAATCTATCTCCTGGA	179
	R- CCAAAATTTCCACTTACTTGTGTTG	
adk	F- TTACTTGGACCTCCAGGTGC	635
	R- TTTCCACTTCCTAAGGCTGC	
atpA	F- TGATGATTTAAGTAAACAAGCTG	674
	R- AATCATGAGTGAAGTCTTCTCC	
dxr	F- GCTACTTTCCATTCTATCTG	525
	R- CCAACTCTTTGTGCTATAAA	
glyA	F- ATAGCTGATGAGGTTGGAGC	625
	R- TTCTAGCCTTAGATTCTTCATC	
recA	F- CAGTAATGAAATTGGGAGAAGC	705
	R- ATTCAGCTTGCTTAAATGGTG	
sodA	F- CCAGTTGTCAATGTATTCATTTC	585
	R- ATAACTTCATTTGCTTTTACACC	
tpi	F- ATGAGAAAACCTATAATTGCAG	640
	R- TTGAAGGTTTAACACTTCCACC	

Table S5. Strain information and GenBank accession numbers of 36 whole-genome sequences for *C. difficile* ST42 and ST203 isolates.

Strain No.	MLST	Source	Accession No.
Z1322HCD0002	203	Human	CP149766
Z1322HCD0027	203	Human	CP149765
Z1322HCD0028	203	Human	CP149762 - CP149764
Z1322HCD0041	203	Human	CP149759 - CP149761
Z1322HCD0043	203	Human	CP149758
Z1322HCD0061	203	Human	CP149753 - CP149757
Z1322HCD0081	203	Human	CP149750 - CP149752
Z1322HCD0094	203	Human	CP149747 - CP149749
Z1322HCD0096	203	Human	CP149746
Z1322HCD0097	203	Human	CP149744 - CP149745
Z1322HCD0107	203	Human	CP149743
Z1322HCD0115	203	Human	CP149732
Z1323HCD0032	203	Human	CP149733 - CP149735
Z1322PCD0001	203	Dog	CP149731
Z1322PCD0023	203	Dog	CP149742
Z1322PCD0024	203	Dog	CP149741
Z1322PCD0028	203	Dog	CP149736 - CP149740
Z1322HCD0006	42	Human	CP149730
Z1322HCD0018	42	Human	CP149727 - CP149729
Z1322HCD0031	42	Human	CP149726
Z1322HCD0033	42	Human	CP149724 - CP149725
Z1322HCD0072	42	Human	CP149719 - CP149723
Z1322HCD0073	42	Human	CP149716 - CP149718
Z1322HCD0087	42	Human	CP149715
Z1322HCD0092	42	Human	CP149713 - CP149714
Z1322HCD0102	42	Human	CP149711- CP149712
Z1323HCD0020	42	Human	CP149693 - CP149694
Z1322PCD0012	42	Dog	CP149689 - CP149692
Z1322PCD0031	42	Dog	CP149708 - CP149710
Z1322PCD0036	42	Dog	CP149706 - CP149707
Z1322PCD0040	42	Dog	CP149705
Z1322PCD0060	42	Dog	CP149703 - CP149704
Z1322PCD0061	42	Dog	CP149702
Z1322PCD0067	42	Dog	CP149701
Z1322PCD0068	42	Dog	CP149698 - CP149700
Z1322PCD0071	42	Dog	CP149695 - CP149697
